# MR Elastography Findings in a Cytotoxic Lesion of the Corpus Callosum (CLOCC): a Case Report with Longitudinal Follow-up

**DOI:** 10.1007/s00062-026-01649-1

**Published:** 2026-04-13

**Authors:** Hannah Mies, Peter Arthur Ringleb, Ralph Sinkus, Katharina Schregel

**Affiliations:** 1 Department of Neuroradiology, University Hospital Heidelberg, Heidelberg, Germany; 2 Department of Neurology, University Hospital Heidelberg, Heidelberg, Germany; 3 Laboratory of Vascular Translation Science, LVTS, U1148, INSERM (National Institute for Health and Medical Research), Paris, France; 4 Department of Radiology, Section of Neuroradiology, Jena University Hospital, Jena, Germany; 5 Department of Neuroradiology, University Hospital Heidelberg, Heidelberg, Germany

## Introduction

Cytotoxic lesions of the corpus callosum (CLOCC) are relatively common MRI findings. However, their pathophysiology remains incompletely understood [1]. A transient cytotoxic edema is hypothesized to cause the observed MRI morphology consisting in a circumscribed central lesion within the splenium of the corpus callosum. These lesions usually are hypointense on T1-weighted (T1w) images and hyperintense on T2-weighted (T2w) or Fluid-Attenuated Inversion Recovery (FLAIR) images and present with restricted diffusion. The so-called boomerang-sign with irregular lesions extending through the splenium in the adjacent parenchyma is less common [2]. CLOCC are considered secondary lesions and identifying their etiology is essential [3]. Potential triggers for the cell-cytokine interaction, which contributes to the development of CLOCC, include medications, malignancies, metabolic disturbances, trauma, and other pathologies [1, 3]. The prognosis of CLOCC depends on the underlying cause but is usually favorable. MRI findings are typically reversible within 1 month, mostly within 1 week after clinical recovery [4]. Despite this, it remains unclear whether CLOCC have a lasting effect on the callosal microstructural integrity as the cell-cytokine interactions can lead to neuronal and microglial dysfunction [3]. Microstructural changes alter viscoelastic tissue properties which can be quantified using magnetic resonance elastography (MRE). MRE is a non-invasive imaging modality to quantify the biomechanical properties of tissues. Based on phase-contrast MRI principles, MRE functions as a quantitative analogue to manual palpation [5]. The technique involves generating mechanical vibrations that induce shear waves traveling through the (brain) tissue, resulting in minimal displacement of tissue components. The analysis of wave propagation enables the assessment of tissue viscoelasticity, commonly referred to as stiffness, which reflects the response of tissue to deformation [6]. MRE has been successfully used both in clinical diagnostics of liver diseases and in experimental research settings. It is conceivable that CLOCC will exhibit localized mechanical softening (i.e., decreased stiffness or altered viscoelastic parameters) during the acute phase, reflecting edema. Over time, as edema resolves, stiffness should normalize. Monitoring stiffness spatially and temporally could thus provide a quantitative biomarker of lesion activity, resolution, and possibly residual microstructural alteration, complementing conventional MRI, diffusion, and spectroscopy.

## Clinical Case

A 66-year-old female patient presented to the neurological emergency department with visual disorders, left-sided hemiparesis and hemiataxia (score on the National Institutes of Health Stroke Scale (NIHSS) 3). The patient’s medical history was notable for hypothyroidism, adequately controlled with medication. She was not currently taking any other medication, except for vitamin supplements, and denied any similar events in the past. An immediately performed cranial computed tomography (CT) showed no evidence of ischemic or inflammatory pathology. A minor stroke was suspected, and the patient was admitted to the stroke unit for further investigation. Thrombolysis was not performed due to a prolonged time window.

**Fig. 1:**
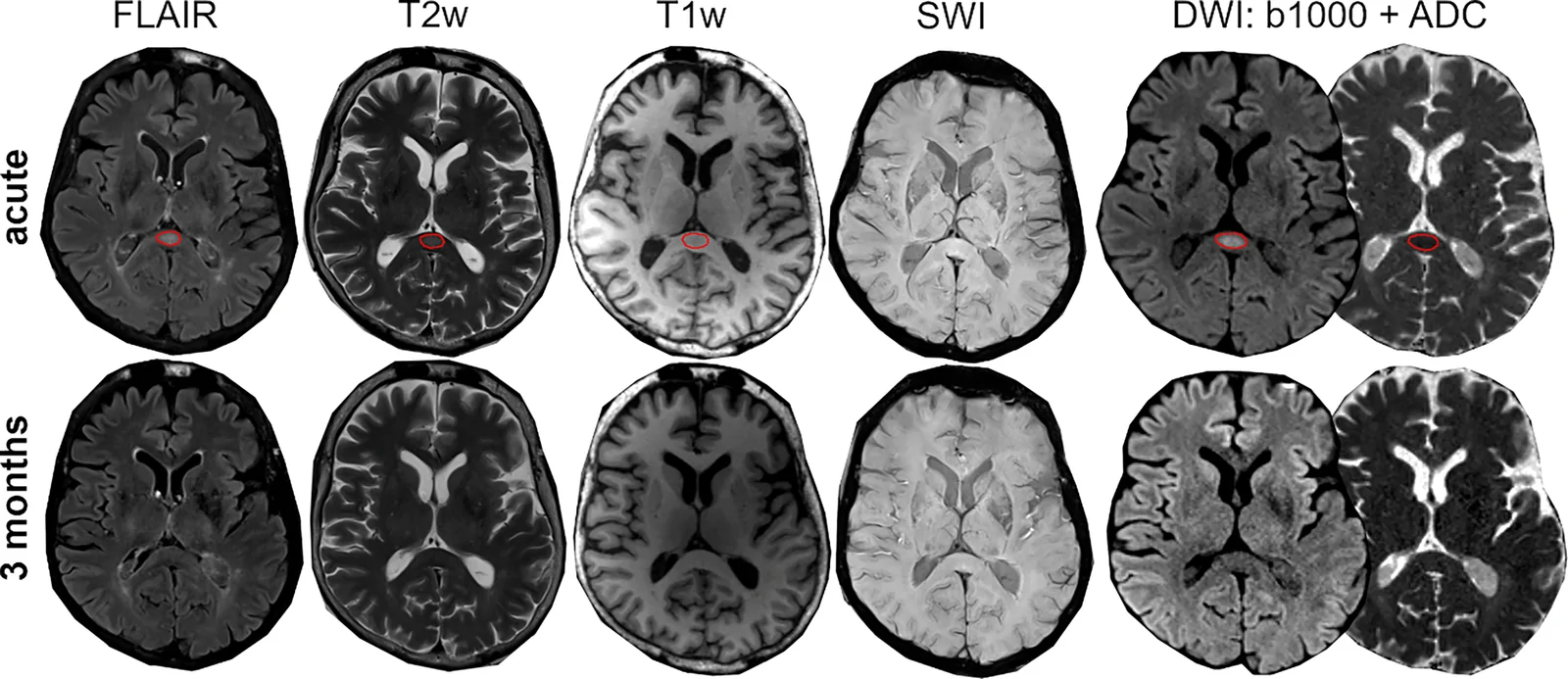
CLOCC on MRI. In the acute phase (top row) a typical FLAIR-/T2w-hyperintense, faintly T1w-hypointense diffusion restricted CLOCC was visible in the midline of the splenium (marked in red). CLOCC had completely resolved after 3 months (bottom row)

As a minor stroke was suspected, the patient was enrolled in a clinical study investigating the biomechanics of ischemic stroke after providing informed consent. This study was performed in line with the principles of the Declaration of Helsinki. Approval was granted by the Ethics Committee of University Heidelberg (permit no. S‑841/2020). As per study protocol, she underwent 3T brain MRI (Prisma fit, Siemens Healthineers, Erlangen, Germany) including 3D T1w MPRAGE, axial T2w TSE, 3D FLAIR as well as susceptibility weighted (SWI), diffusion weighted (DWI) and arterial spin labeling (ASL) perfusion imaging. Moreover, three-dimensional generalized multi-shot gradient-recalled echo (3D GRE Ristretto [7]) MRE was acquired using a gravitational transducer set-up [8] at a vibration frequency of 50 Hz. The following acquisition parameters were applied: repetition time (TR)/echo time (TE): 100/9.84 ms; flip angle: 25°; field of view read-out: 192 mm; slice thickness: 1.5 mm; number of slices: 8; number of wave phases: 8; strength of motion-encoding gradients: 20 mT/m; Hadamard encoding. MRE data reconstruction was realized according to published algorithms [9, 10] using dedicated in-house software (ROOT environment, CERN; Meyrin, Switzerland). Processing included per slice unwrapping and alignment of phase data followed by calculation of an 8‑point Fourier transform. The first harmonic amplitude was used to represent the displacement amplitude. Compression waves were removed using the curl of the displacement data. Spatial derivatives were calculated and used to inverse the Helmholtz equation. Finally, color-coded elastograms were calculated and used for further analyses. Quality of the MRE data was assessed using nonlinearity, a metric informing about the the quality of the temporal sinusoidal motion of a voxel, and the ratio of the curl over the divergence, which is a pertinent marker to gauge the quality of the shear wave quantification. Data quality was deemed acceptable when mean nonlinearity was < 25% and the ratio of the curl over the divergence was > 2 as previously described [11]. All datasets met these quality metrics (mean and between-subject 95% confidence interval (CI) of nonlinearity 19.52%, 95% CI [11.06, 27.98] and |curl|/|divergence| 7.08, 95% CI [5.69, 8.45]). Elastograms were exported in NifTI format. Combined analysis of MRI and MRE data was realized using open-source 3D slicer software (3D Slicer for Windows, version 5.10.0, www.slicer.org [12]). Elastograms as well as the MRI images were co-registered to the respective 3D T1w MPRAGE sequence of each subject applying a linear rigid transform. Manual segmentation of CLOCC was performed on FLAIR images. Moreover, regions of interest (ROI) of comparable size were placed in the splenium of control subjects as well as in the unaffected corpus callosum of the patient. This “unaffected corpus callosum”-ROI in the patient encompassed non-CLOCC regions of the splenium as well as additional callosal segments. To account for potential systematic biomechanical differences between the splenium and the remainder of the corpus callosum, we segmented analogous “unaffected corpus callosum”-ROIs in the control group. Mean values of the MRE-parameters were calculated in CLOCC- as well as control-ROIs and compared. In addition, the mid-splenial-ROI was compared to the “unaffected corpus callosum” ROIs in the control group. The MRI of the patient showed a typical CLOCC, presenting as a circumscribed oval hyperintensity on T2w and FLAIR with faint T1w-hypointensity and restricted diffusion in the midline of the splenium (Fig. 1). Biomechanical properties within the CLOCC region were quantitatively compared to unaffected regions of the patient’s corpus callosum as well as to those of ten subjects without CLOCC (controls) also participating in the study. The control group consisted in patients presenting with neurological symptoms consistent with stroke that were admitted to the stroke unit of Heidelberg University Hospital (mean age ± standard deviation (SD): 69 ± 14.31 years; 8 male, 2 female; mean NIHSS scores ± SD at admission 5.2 ± 3.16 and at discharge 0.6 ± 0.97). In this cohort, seven subjects demonstrated an acute infarct, as detected on DWI and FLAIR sequences. In the remaining three patients, no ischemic lesion was identified on MRI, presumably reflecting symptoms attributable to a transient ischemic attack or effective reperfusion following timely intravenous thrombolysis. Normal appearance of the corpus callosum was confirmed in all subjects on MR imaging. Follow-up imaging in the patient with CLOCC was performed after three months. At that time, the patient’s symptoms as well as the MRI findings had completely resolved (Fig. 1). A specific therapy had not been initiated, as the underlying cause remained unclear. The patient’s vitamin supplementation was discussed as a potential contributing factor.

## Results and Discussion

For the biomechanical analysis, we focused on the complex shear modulus |G*| (stiffness) and its elastic ($G^{\prime}$) and viscous ($G^{\prime\prime}$) components ($| G^{*}| =\sqrt{G^{\prime2}+G^{\prime\prime2}}$) as well as the phase angle Y. The latter describes the relative contributions of elasticity and viscosity to the shear modulus and is calculated as $Y=\frac{2}{\pi }\,\mathrm{atan}(\frac{G^{\prime\prime}}{G^{\prime}})$; a lower phase angle thus indicates predominantly elastic tissue properties, whereas higher values reflect more viscous behavior.

**Fig. 2:**
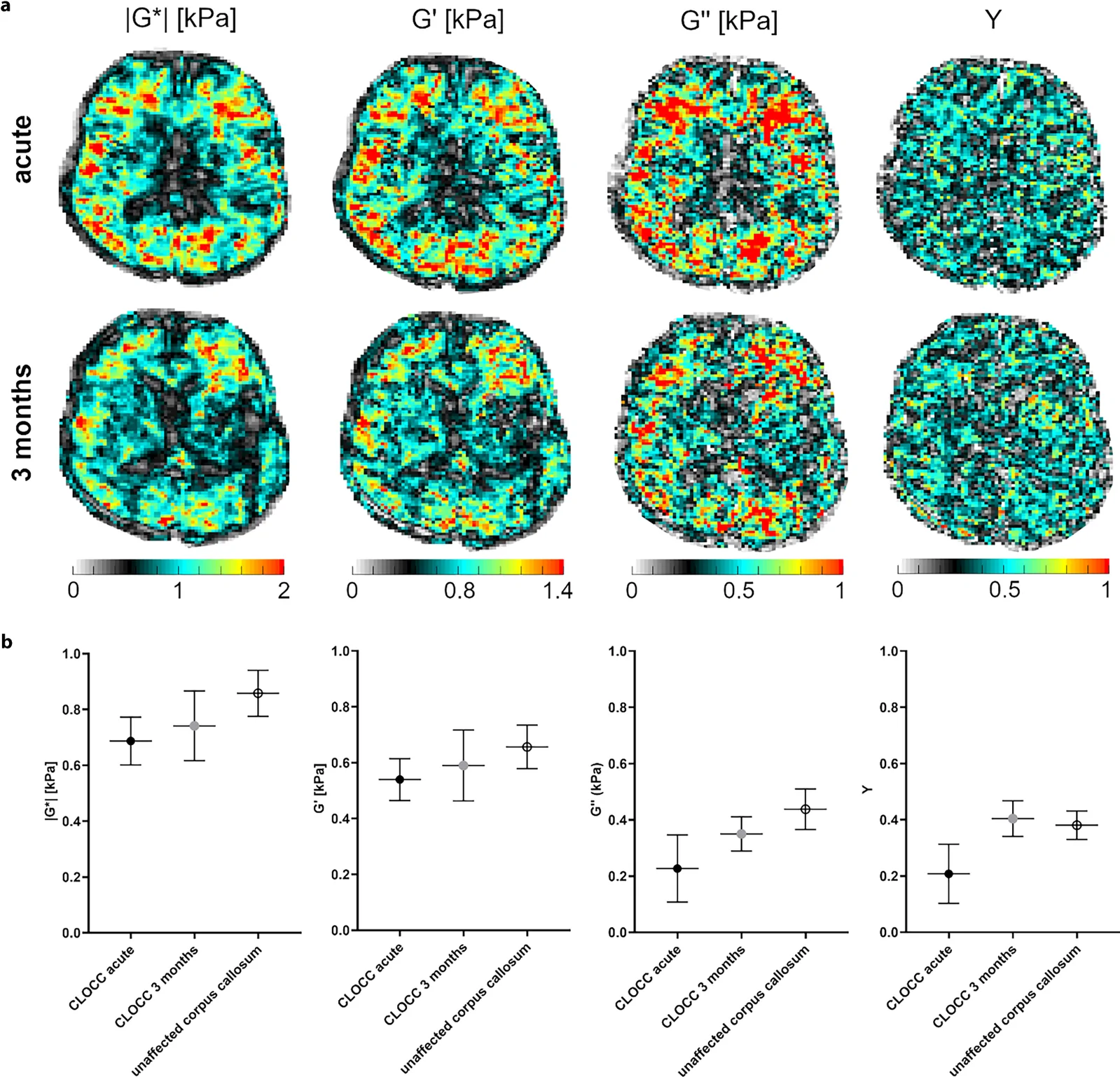
CLOCC on MRE. In the acute phase (**a** top row; **b** black dots), CLOCC are softer and less viscous compared to unaffected callosal tissue (**b** white dots). After 3 months (**a** bottom row; **b** grey dots), the biomechanical changes of CLOCC are only partially reversible, indicating ongoing microstructural remodeling. Mean and 95% CI of within ROI deviation are shown in (**b**)

All biomechanical parameters were reduced in acute CLOCC compared to unaffected areas of the corpus callosum within the same patient [Table 1; Fig. 2; mean values and 95% CI of within ROI-deviation CLOCC vs. unaffected corpus callosum: |G*| 0.69 kPa, 95% CI [0.60, 0.77] vs. 0.86 kPa, 95% CI [0.76, 0.94]; G′ 0.54 kPa, 95% CI [0.46, 0.61] vs. 0.66 kPa, 95% CI [0.58, 0.73]; G″ 0.23 kPa, 95% CI [0.11, 0.35] vs. 0.44 kPa, 95% CI [0.37, 0.51]; Y 0.21 95% CI [0.10, 0.31]vs. 0.38 95% CI [0.33, 0.43]]. The reduction of |G*| and G′ is expected given the presumed cytotoxic edema of CLOCC. Increased water content and edema tend to soften brain tissue which has been shown in various studies investigating neuroinflammation in preclinical models and in patients [13–15]. However, the reduction of viscosity and the phase angle cannot be explained by edema alone. Viscosity expresses the strain rate of tissue and, similar to the phase angle, is a measure of the energy dissipated in tissue as a result of mechanical friction [5, 16]. Previous studies demonstrated that a decrease of these parameters is associated with alterations of a material’s or the brain’s network structure [17, 18]. Thus, the observed reduction of viscosity and the phase angle suggest additional microstructural alterations within CLOCC.

**Table 1:** Overview of key results.

Subject	Region	|G*| (in kPa)	G′ (in kPa)	G″ (in kPa)	Y (in kPa)
Patient	CLOCC acute	0.69	0.54	0.23	0.21
	CLOCC 3 months follow-up	0.74	0.59	0.35	0.40
	Unaffected corpus callosum	0.86	0.66	0.44	0.38
Control 1	Midline splenium	0.54	0.48	0.21	0.27
Control 2		0.79	0.53	0.48	0.46
Control 3		1.01	0.60	0.68	0.50
Control 4		0.74	0.52	0.38	0.43
Control 5		0.40	0.34	0.15	0.28
Control 6		1.07	0.75	0.45	0.37
Control 7		0.82	0.60	0.32	0.33
Control 8		0.58	0.49	0.23	0.23
Control 9		0.93	0.66	0.47	0.37
Control 10		0.71	0.60	0.27	0.26
Control 1	Remaining corpus callosum	0.77	0.61	0.37	0.32
Control 2		0.96	0.73	0.51	0.38
Control 3		1.11	0.81	0.62	0.43
Control 4		0.89	0.65	0.43	0.42
Control 5		0.74	0.53	0.43	0.42
Control 6		0.93	0.77	0.36	0.27
Control 7		1.03	0.72	0.51	0.42
Control 8		1.08	0.76	0.55	0.38
Control 9		0.92	0.65	0.49	0.43
Control 10		0.67	0.50	0.35	0.44

**Fig. 3:**
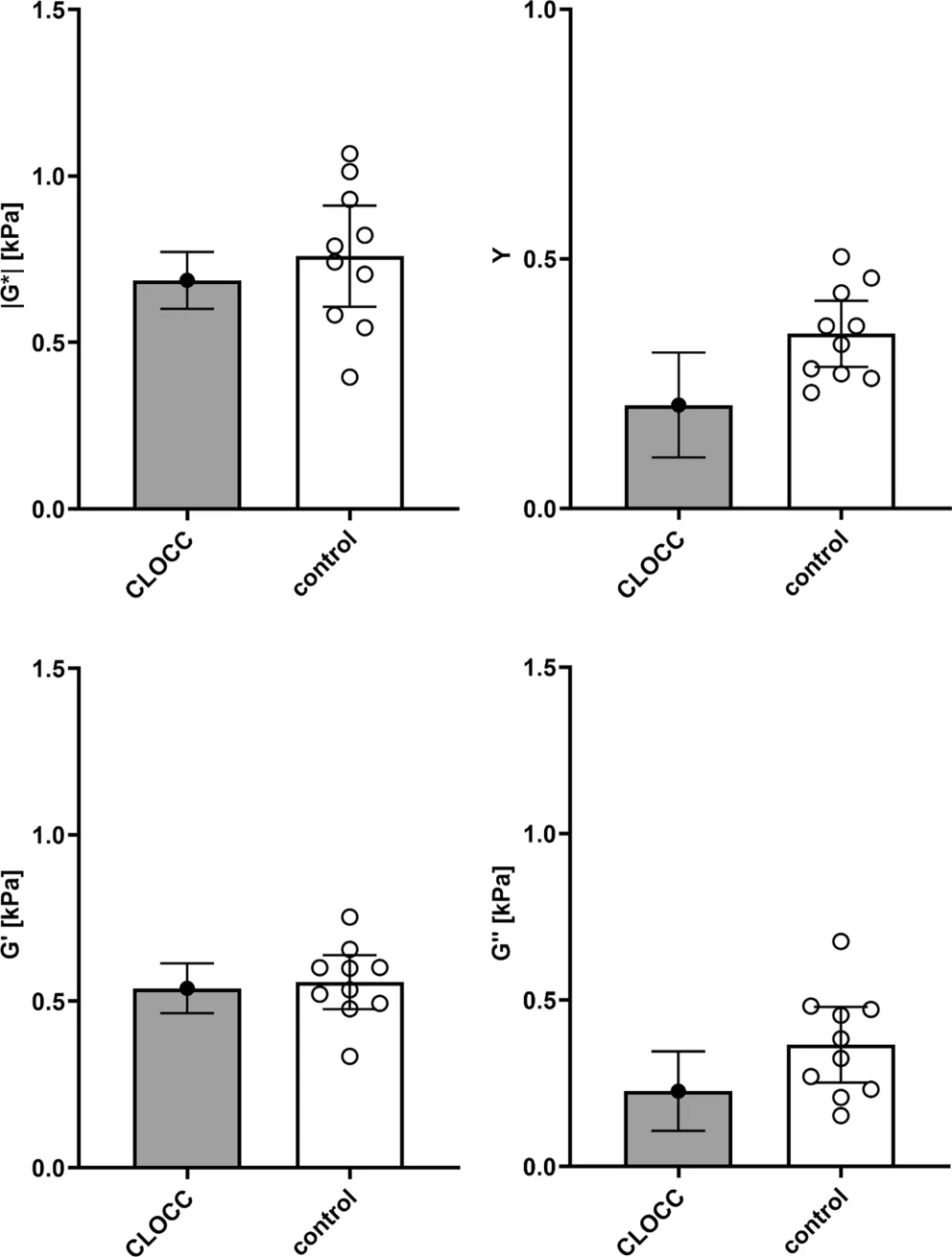
Biomechanics of acute CLOCC compared to the splenium of control subjects. MRE detects focal biomechanical changes related to CLOCC despite background deviations in splenium biomechanics of control subjects. Mean and 95% CI of within ROI- (CLOCC) and between subject-deviation (controls) are shown

When comparing the biomechanical properties of CLOCC to corresponding regions in the splenium of control subjects, mean values of both phase angle and viscosity were lower in CLOCC; however, the 95% CI overlapped, suggesting a trend rather than a statistically significant difference. Stiffness and elasticity were comparable between CLOCC and unaffected splenium [Table 1; Fig. 3; mean values and between-subject 95% CI of controls: |G*| 0.76 kPa, 95% CI [0.61, 0.91]; G′ 0.56 kPa, 95% CI [0.48, 0.64]; G″ 0.36 kPa, 95% CI [0.25, 0.48]; Y 0.35, 95% CI [0.28, 0.42]]. The corpus callosum has a well-characterized mechanical baseline. The callosal white matter is relatively stiff compared to surrounding tissue in healthy subjects [19]. Our analysis shows that MRE can indicate focal biomechanical changes related to CLOCC over background variations.

In control subjects, the “unaffected corpus callosum” demonstrated significantly higher stiffness and elasticity than the midline splenium in controls (Table 1**; **mean |G*| and G’ of unaffected corpus callosum vs. midline splenium 0.91 kPa, 95% CI [0.80, 1.01] vs. 0.76 kPa, 95% CI [0.61, 0.91], *p* = 0.03 and 0.67 kPa, 95% CI [0.60, 0.74] vs. 0.56 kPa, 95% CI [0.48, 0.64], *p* = 0.02). Viscosity and phase angle were comparable between the regions (mean G″ and Y of unaffected corpus callosum vs. midline splenium 0.46 kPa, 95% CI [0.40, 0.53] vs. 0.37 kPa, 95% CI [0.25, 0.48], *p* > 0.05 and 0.39, 95% CI [0.35, 0.43] vs. 0.35, 95% CI [0.28, 0.42], *p* > 0.05).

Thus, there is regional biomechanical variation in the corpus callosum which manifests in stiffness and elasticity but not in viscosity and phase angle. Even though the corpus callosum appears homogeneous on MRI, it is histologically heterogeneous. Light-microscopic evaluation revealed that thin fibers are most dense in the anterior corpus callosum, decrease toward the posterior midbody, and increase again in the splenium, with a localized reduction at the posterior pole, whereas large-diameter fibers show a complementary distribution, peaking in the posterior midbody with a localized increase at the posterior pole [20]. It is conceivable that these regional microstructural differences within the corpus callosum preferentially affect tissue stiffness/elasticity rather than phase angle/viscosity. The MRE-derived elasticity primarily reflects the tissue’s capacity to store mechanical energy and is strongly influenced by structural features such as axonal packing density, fiber diameter, and myelination. In contrast, viscosity is thought to be more closely related to dissipative processes, including fluid-related friction and cellular or extracellular matrix interactions, which may be less sensitive to variations in axonal caliber and fiber organization. Consequently, microstructural heterogeneity of callosal fibers could plausibly manifest as regional differences in stiffness and elasticity without corresponding changes in phase angle and viscosity. Although these regional differences in callosal biomechanics are of interest, they are unlikely to have substantially influenced our observations. The biomechanical properties of CLOCC were compared with those of an anatomically and architecturally comparable midline region of the splenium in control subjects. Furthermore, mean values of phase angle and viscosity, parameters considered less sensitive to underlying fiber architecture, tended to be lower in CLOCC than in controls, arguing against a relevant confounding effect of callosal microstructural heterogeneity.

Within three months following the diagnosis of CLOCC, stiffness, elasticity and viscosity increased without reaching the values of unaffected callosal tissue [Fig. 2; mean values and 95% CI of within ROI-deviation CLOCC 3 months: |G*| 0.74 kPa, 95% CI [0.62, 0.87]; G’ 0.59 kPa, 95% CI [0.46, 0.72]; G″ 0.36 kPa, 95% CI [0.29, 0.41]; Y 0.40, 95% CI [0.34, 0.47]]. Only the phase angle of the CLOCC region approximated unaffected callosal tissue at this time point [Fig. 2; mean Y CLOCC 3 months 0.40, 95% CI [0.34, 0.47]]. This indicates only partial restitution of splenial biomechanics 3 months after onset of CLOCC.

Our analysis suggests that CLOCC exerts a localized impact on cerebral microstructure and, consequently, on the biomechanical properties of brain tissue. These alterations can be quantitatively assessed using MRE. The observed initial intra- and interindividual reduction in tissue stiffness, elasticity and viscosity within CLOCC, implies that, beyond cytotoxic edema, additional pathological alterations such as further cellular changes and/or extracellular matrix remodeling could be involved. Longitudinally, only partial recovery of biomechanical parameters is evident.

## Conclusion

To our knowledge, this case report presents the first biomechanical characterization of CLOCC. Further investigations are required to validate our observations and enable comparative analyses across a larger cohort. Nevertheless, the present case suggests that MRE may serve as a promising tool in advancing our understanding of the pathophysiology of CLOCC.

## Data Availability

All data used for this study is presented in the manuscript.
